# The DnaA Protein Is Not the Limiting Factor for Initiation of Replication in *Escherichia coli*

**DOI:** 10.1371/journal.pgen.1005276

**Published:** 2015-06-05

**Authors:** Ingvild Flåtten, Solveig Fossum-Raunehaug, Riikka Taipale, Silje Martinsen, Kirsten Skarstad

**Affiliations:** Department of Cell Biology, Institute for Cancer Research, Oslo University Hospital, Oslo, Norway; Agency for Science, Technology and Research (A*STAR), SINGAPORE

## Abstract

The bacterial replication cycle is driven by the DnaA protein which cycles between the active ATP-bound form and the inactive ADP-bound form. It has been suggested that DnaA also is the main controller of initiation frequency. Initiation is thought to occur when enough ATP-DnaA has accumulated. In this work we have performed cell cycle analysis of cells that contain a surplus of ATP-DnaA and asked whether initiation then occurs earlier. It does not. Cells with more than a 50% increase in the concentration of ATP-DnaA showed no changes in timing of replication. We suggest that although ATP-DnaA is the main actor in initiation of replication, its accumulation does not control the time of initiation. ATP-DnaA is the motor that drives the initiation process, but other factors will be required for the exact timing of initiation in response to the cell’s environment. We also investigated the *in vivo* roles of *datA* dependent DnaA inactivation (DDAH) and the DnaA-binding protein DiaA. Loss of DDAH affected the cell cycle machinery only during slow growth and made it sensitive to the concentration of DiaA protein. The result indicates that compromised cell cycle machines perform in a less robust manner.

## Author Summary

Cell cycle regulation of the bacterium *Escherichia coli* has been studied for many years, and its understanding is complicated by the fact that overlapping replication cycles occur during growth in rich media. Under such conditions cells initiate several copies of the chromosome. The active form of the CDC6-like DnaA protein is required for initiation of synchronous and well-timed replication cycles and is in a sense the motor of the cell cycle machine. It has long been debated whether it is the accumulation of enough ATP-DnaA that triggers initiation and determines the replication frequency. In this work we have constructed a strain where the “accumulation of ATP-DnaA triggers initiation” model could be tested. Our results indicate that this model requires some modification. We suggest that cell cycle regulation in *E*. *coli* has similarities to that of eukaryotes in that origins are “licensed” to initiate by a cell cycle motor and that the precise timing depends on other signaling.

## Introduction

The ORC- and CDC6-like prokaryotic initiator protein DnaA has been studied extensively for many years, but it is still not clear whether the protein contributes to actual regulation of the initiation of replication or whether it works as a cell cycle motor which “licenses” initiation at regular intervals. In *E*.*coli* the DnaA protein causes strand opening and recruits the helicase and is thus the key contributor to initiation of replication [1,2]. The DnaA protein, bound to ATP or ADP [3], binds to specific DnaA binding sites within the origin [4–6]. High-affinity binding sites can bind both forms of the DnaA protein [3–6] while low-affinity sites bind only the ATP-bound form [7]. The high-affinity boxes are most likely bound by DnaA throughout the cell cycle [8], while binding to the “last” low-affinity sites has been suggested to trigger the initiation process at a time when the ATP-DnaA level has reached a threshold concentration [9]. Formation of a DnaA oligomer in the origin region causes the unwinding of the DNA in the AT-rich region and formation of the open complex [1,3]. This process is probably facilitated by transcription by RNA polymerase [10–13] and by DiaA, a DnaA-binding protein that has been shown to promote formation of ATP-DnaA complexes at *oriC* and stimulate *oriC* unwinding *in vitro* [14–16].

The DnaA protein also has a role as a transcription factor regulating its own transcription [17–20] and the transcription from several other promoters (see [21] for review) some of which are located close to or within the origin region [22]. More recently it was shown to interact directly with the RNA polymerase and to affect the transcription from the *gidA* promoter, which is situated right next to the origin [23].

The *datA* site is a 1 kb DNA sequence with five well conserved DnaA-boxes [24] and several weak DnaA-boxes [25]. The *datA* region has been thought to bind a large amount of the DnaA protein [24,26], and thereby contribute to titrate the DnaA protein away from the origin. However, recently it was shown that *datA*, together with the IHF protein, has the ability to stimulate the hydrolysis of the DnaA-bound ATP and thereby inactivate the DnaA protein in a process called *datA* dependent inactivation of DnaA (DDAH) [27]. The level of ATP-DnaA is also affected by the RIDA (Regulatory Inactivation of DnaA) process, where the Hda protein together with the β-clamp of the polymerase stimulates the hydrolysis of the ATP bound to DnaA [28]. Mutations which block RIDA are lethal because they lead to massive over-initiation [29,30] whereas deletion of *datA* has minor impact on cell growth [26,31] indicating that RIDA is the more important of the two DnaA inactivation systems. *De novo* synthesis, DARS (DnaA Reactivating Sequence) sites and possibly acidic phospholipids contribute to the regeneration of the active ATP-bound form of the DnaA protein (see [32] for review).

In several earlier studies with over-expression of the DnaA protein, it was shown that a surplus of DnaA in the cells led to excess initiations and reduced initiation mass. It was therefore concluded that the DnaA protein was the factor limiting the initiation frequency [33–38]. However, in many cases the increase in the DNA/mass after the overproduction of DnaA was low [33,35,38,39] or no increase was observed at all [40], results that contradict this conclusion. It has also been shown that cells grown under different conditions can initiate with widely different amounts of DnaA available per origin [41] and that cells grown in minimal medium supplemented with acetate have more DnaA available per origin than cells grown in richer media [23]. Recently it was shown that cells with a reduced cellular amount of DnaA protein (caused by an elevated level of SeqA, which negatively affects the *dnaA* transcription) have no problem initiating replication [42]. In addition to this it has been shown that the presence of additional copies of *oriC* on a high copy number plasmid, which would titrate DnaA away from the chromosomal origin, does not change the timing of initiation [43]. Thus, it is hard to understand that the total amount of DnaA is limiting for initiation of replication. Instead, it is more reasonable to assume that even though enough DnaA must be present at the time of initiation, the accumulation of DnaA alone does not trigger the initiation of replication.

In an attempt to clarify the role of the DnaA protein in the timing of initiation of replication we have performed flow cytometry analysis of cells containing one or several extra copies of the *dnaA* gene and cells with minor changes in the cell cycle machinery. We found that a two-fold elevation in the DnaA concentration does not affect the timing of initiation of replication in any of the growth conditions tested. This argues that the DnaA protein, although it constitutes the key feature of the cell cycle motor, is not the limiting factor for precise timing of initiation of replication.

## Results

### A two-fold increase in DnaA concentration does not affect the timing of replication initiation

**Fig 1:**
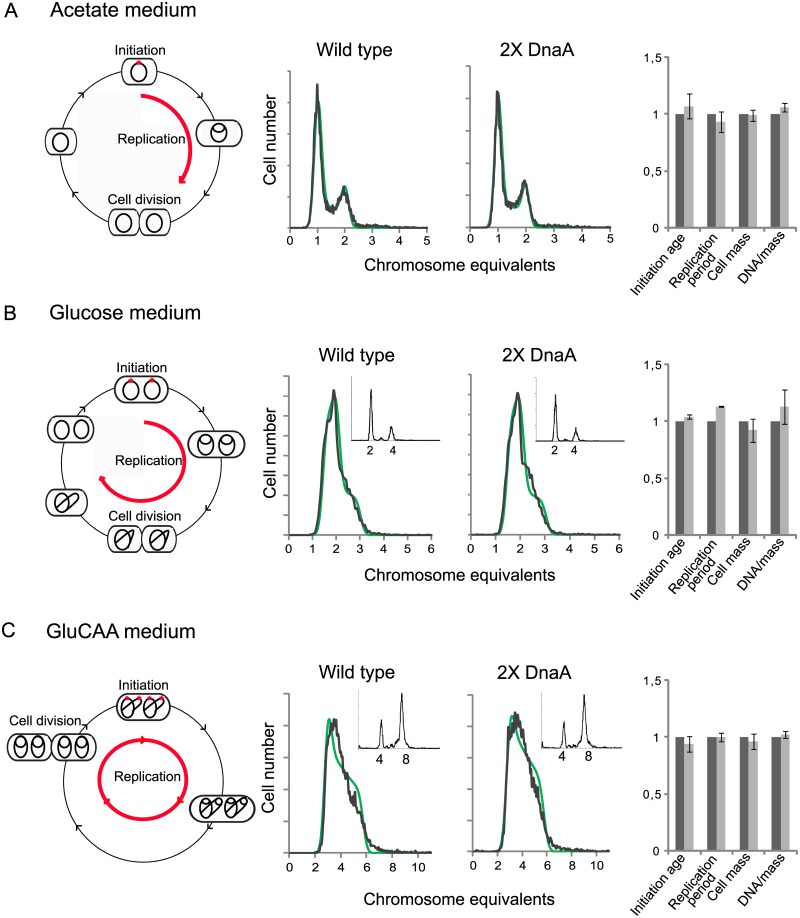
Cell cycle parameters are not changed in cells with a two-fold DnaA concentration. Exponentially growing cells were analyzed by flow cytometry and cell cycle parameters were calculated. The first (leftmost) panels illustrate replication patterns of wild type (MG1655) cells grown in acetate medium (A), glucose medium (B) and GluCAA medium (C). Cells with chromosomes (black lines) were drawn schematically to show the number of replication forks at different stages of the cell cycle. In acetate grown cells initiation of replication occurred at one origin (red dot) and the replication period (red arrow) was completed within one generation (A). In the glucose and GluCAA grown cells initiation of replication occurred at two and four origins, respectively (B-C). The second and third panels (A-C) show representative DNA histograms of exponentially growing MG1655 and IF72 cells with run-out DNA histograms inserted for (B) and (C). The theoretical curves of the best fit simulation are shown in green. The chromosome equivalents are shown on the abscissa and the number of cells on the ordinate. 10000 cells were measured and one tick on the ordinate represents 100 cells. The calculated values for initiation age, replication period, cell mass and DNA/mass relative to the values for the wild type are shown in the bar histograms in panel four (A-C). The values are an average of three or more experiments and the error bars represent the standard deviation. For a more detailed description of the cell cycle parameters see S2 Fig. The length of the replication period was also determined with quantitative PCR of the *oriC*/*ter* ratio for the cells grown in GluCAA medium (S6 Table).

In order to investigate the effects of a moderate increase in the DnaA concentration on initiation of DNA replication, cells with one extra copy of the *dnaA* gene under control of its own promoter, leading to a two-fold increase in the DnaA concentration (Table 1 (IF72) and S1 Fig) were analyzed and compared to the parent cells (Fig 1). The cells were grown in minimal medium supplemented with acetate, glucose or glucose and casamino acids (GluCAA), which led to generation times of about 4 hours, 70 minutes and 30 minutes, respectively (S1 Table).

**Table 1:** DnaA concentrations in DnaA overproducing cells, Δ*datA* cells and DiaA overproducing cells.

Strain	Medium	Relative DnaA concentration^1)^
IF72	Acetate	1.79 ± 0.12
IF72	Glucose	1.52 ± 0.08
IF72	GluCAA	1.72 ± 0.25
MOR90	Acetate	34.7 ± 1.90
MOR90	Glucose	10.3 ± 0.65
MOR90	GluCAA	11.4 ± 1.69
MOR177	Acetate	0.88 ± 0.15
MOR177	Glucose	1.01 ± 0.17
MOR177	GluCAA	0.81 ± 0.12
IF97	GluCAA	1.20 ± 0.12

^1)^ ng DnaA per μg cell extract. The numbers are relative to the wild type or wild type containing the empty plasmid. Measured by imunoblotting.

± represents the standard deviation

DNA and mass distributions of the cells were obtained by flow cytometry analysis, and were found to give similar results for cells with wild type and two-fold extra DnaA in all three media (Fig 1 and S1 Table). When all cells in a population grow exponentially (with the same generation time and the same replication pattern), it is possible to use the information in the DNA histograms to calculate the cell-cycle parameters (initiation age and replication period). This calculation was done in an excel based simulation program [44] (Fig 1 and S1 Table).

Wild type cells growing slowly in acetate medium initiated at one origin and had two replication forks in the replication period. The fraction of replicating cells (the population of cells containing between one and two chromosome equivalents in the DNA histogram (Fig 1A, middle panels)) was 25–29%. This yielded a replication period of about 80 min (red arrow in Fig 1A, leftmost panel). Cells growing more rapidly in glucose medium were found to initiate replication at two origins in the mother cell before cell division (Fig 1B, leftmost panel) and cells contained through the cell cycle either two whole chromosomes or one or two partially replicated chromosomes (Fig 1B, middle panels). Cells growing faster still, in GluCAA medium, exhibited overlapping replication cycles and initiated replication in the “grandmother” generation at four origins (Fig 1C, leftmost panel). These cells had DNA contents that ranged from three to six chromosome equivalents (Fig 1C, middle panels). To aid the determination of the cell cycle parameters a part of the cell cultures grown in glucose and GluCAA medium were treated with rifampicin and cephalexin to obtain so-called replication run-out DNA histograms (see Materials and Methods). In the drug treated cells (Figs 1B and 1C, small histograms) all ongoing replication was allowed to finish, but new initiations and cell division were inhibited. The number of fully replicated chromosomes then represents the number of origins present in the cells at the time of drug addiction [45]. All origins in a cell are initiated in synchrony and therefore the cells will end up with numbers of chromosomes which are exponential multiples of two (2^n^) after drug treatment [46]. If control of initiation is compromised this can often be seen as asynchronous initiations, i.e. a chromosome number different from 2^n^. Here, the cells with two-fold extra DnaA were found to contain the same number of origins as the wild type cells and initiation occurred in synchrony (Figs 1B and 1C, small histograms). Also the average age at initiation, duration of the replication period, average cell mass and the DNA concentration were found to be essentially the same as in wild type cells in all media tested (Fig 1, rightmost panels, S1 Table and S2 Fig). These results indicate that the amount of DnaA in the cell is not likely to be the factor limiting initiation of replication under the growth conditions tested.

It has previously been shown that a large excess of DnaA causes more frequent initiation (see below), and that some replication forks collapse and fail to extend beyond the immediate area flanking *oriC* [47]. To check that this does not occur in our situation with two-fold extra DnaA, we performed marker frequency analysis of the *oriC* and *Ter* region. If initiation had occurred but a failure of forks to extend beyond the immediate area flanking *oriC* was a problem, this would have shown up as an increase in the *oriC*/*ter* ratio in the constructed strain compared to the wild type. It does not (S6 Table). Thus, we conclude from our results that there are no extra initiations in the cells with two-fold extra DnaA.

It has been shown that only the ATP-form of DnaA is active and capable of performing strand opening *in vitro* [48]. Therefore, one explanation of the above result could be that the amount of ATP-DnaA is unchanged in the cells with a two-fold DnaA concentration, i.e. that the ratio of ATP- to ADP-DnaA is much lower than normal. We therefore investigated the ratio of ATP-DnaA to ADP-DnaA in the wild type cells and the cells with two-fold concentration of DnaA. We then found that the ratios were about the same in the two strains. This means that there is an increase of both forms of the DnaA protein and that the level of ATP-DnaA is indeed significantly higher in the cells with a two-fold DnaA concentration (Table 2 and S3 Fig). Thus, the result indicates that in cells with a substantial surplus of ATP-DnaA, timing of initiation is controlled in the same way as in wild type cells, and that the controller is not the amount of ATP-DnaA.

**Table 2:** Percentage of ATP-DnaA protein in cells with 2 x DnaA.

Strain	DnaA concentration ^1)^	DnaA molecules per cell^2)^	Percent ATP-DnaA	Number of DnaA molecules in ATP-form	Relative ATP-DnaA concentration
MG1655	1	1000	41 ± 2	410	1
IF72	2	2000	37 ± 2	740	1.8

^1)^ Measured by western blot

^2)^ Number derived from [24]

### Cells with a surplus of ATP-DnaA are otherwise the same as the wild type cells

Before accepting the above result we found that it would be important to check that other factors were not “compensating”, i.e. that the production of extra DnaA did not lead to two changes in timing of replication which cancelled each other. We therefore checked the expression of genes possibly affected by the change in the concentration of DnaA. The *dnaN* gene, encoding the β clamp of the DNA polymerase, is located in the same operon as the *dnaA* gene and its expression is therefore, at least partially, dependent on the *dnaA* promoter [49]. Because this promoter is auto regulated by the DnaA protein there was a possibility that the extra DnaA in the cells repressed this promoter leading to a decreased level of the β clamp. If so, it could be an explanation of the lack of effect of the two-fold extra DnaA. We therefore quantified the level of β clamp in the wild type cells and cells with two-fold extra DnaA by western blotting. No changes in the level of β clamp were detected (S2 Table). We also checked whether the two-fold extra DnaA would give an effect if accompanied by extra β clamp protein. Wild type cells and cells with two-fold extra DnaA were transformed with a plasmid containing the *dnaN* gene under control of an inducible promoter. Induction with a low level (30μM) IPTG led to levels of DnaN that were between 40 and 80% above wild type level. When these cells were analyzed using flow cytometry no significant changes in the initiation age or the C-period were observed in either wild type or the cells with two-fold extra DnaA (S3 Table). We therefore conclude that the lack of change in the timing of initiation in the cells with two-fold extra DnaA was not due to a reduced level of β clamp.

Since the DnaA protein has also been shown to regulate several other genes in addition to its own and *dnaN* (see [21] for review), we also performed RNA sequencing to investigate differences in the transcription between the wild type cells and the cells with two-fold extra DnaA (see Materials and Methods). This generated a list of 4692 transcripts (S7 Table) out of which four genes were differentially expressed in the two strains (Table 3). One of these was the DnaA protein itself confirming the elevated transcription of this gene. This also functions as a positive control of the sequencing experiment. Two of the other genes that were up-regulated in the cells with two-fold extra DnaA are situated right next to the λ *att* site where the extra copy of the *dnaA* is inserted on the chromosome (See Materials and Methods). Thus, the increased transcription of these two genes is likely to be an effect of the increased local transcription in their surroundings rather than a result of the extra DnaA in the cell. The fourth gene, *pka*, which has a slightly elevated transcription level encodes an protein lysine acetyltransferase. The roles of acetylation in prokaryotes are not very well known, but one function is apparently to make the cells more tolerant towards environmental stress [50]. We think that it is unlikely that the slight elevation in the level of this protein affects the timing of initiation of replication in the cells with two-fold extra DnaA. The RNA sequencing also confirmed the unchanged level of the *dnaN*, encoding the β clamp of the DNA polymerase, as well as other genes that might influence the initiation process such as *mioC*, *gidA*, *seqA* and *nrdAB* [21].

**Table 3:** Differentially expressed genes in cells with 2 x DnaA vs wild type.

gene	product	Expression MG1655	Expression IF72	Fold change	q value MG1655 vs IF72
*pka*	protein lysine acetyltransferase	103	171	1.66	0.0086
*dnaA*	chromosomal replication initiator protein	61	165	2.70	0.0000
*bioA*	7,8-diaminopelargonic acid synthase	32	194	6.06	0.0000
*ybhB*	kinase inhibitor homolog, UPF0098 family	117	1087	9.29	0.0000

### A large increase in the DnaA concentration leads to excessive and asynchronous initiations

**Fig 2:**
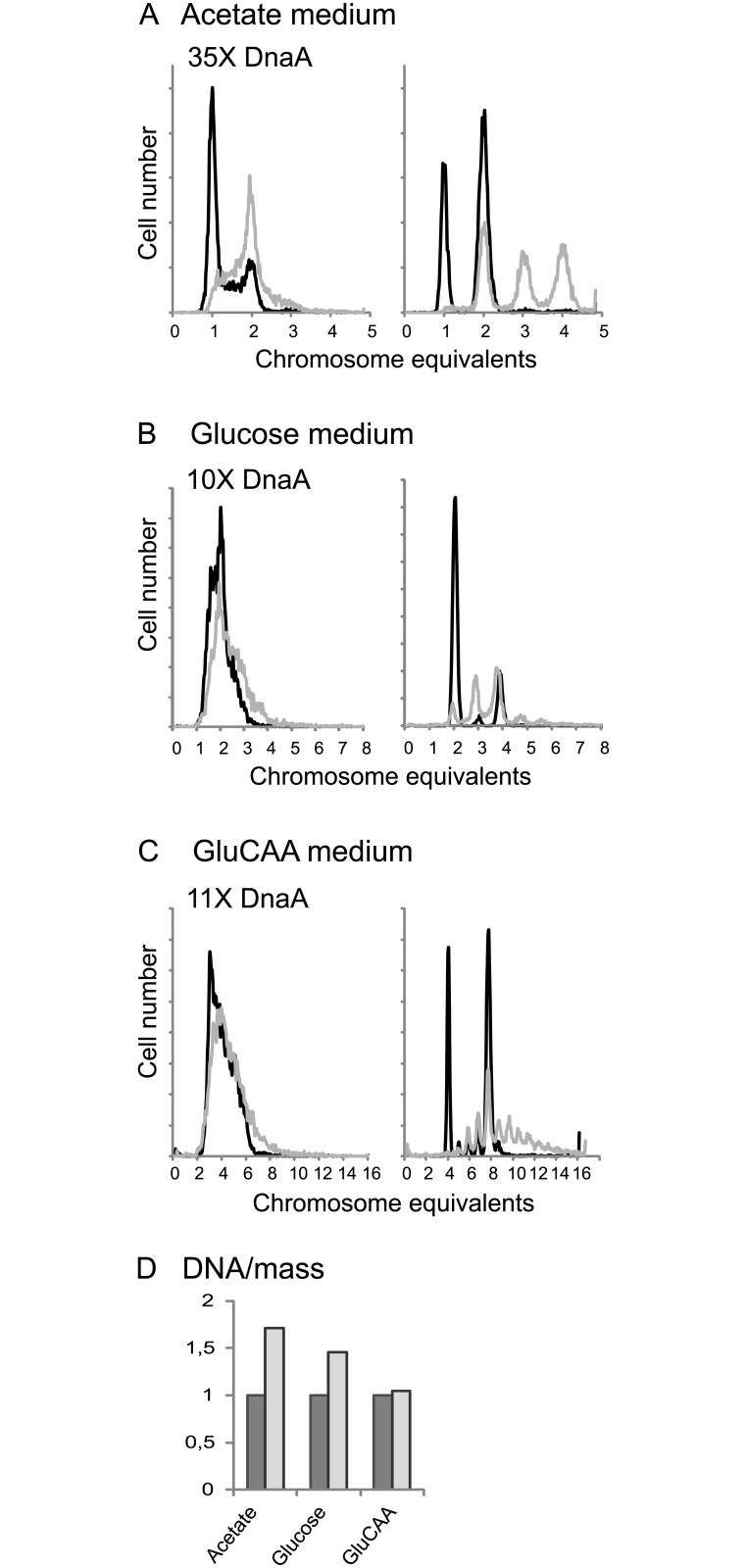
A large excess of DnaA protein changes the cell cycle. Wild type cells (containing the empty vector, IF26) and cells with a large excess of DnaA protein (MOR90) were grown in acetate medium (A), glucose medium (B) and GluCAA medium (C) and investigated by flow cytometry. DNA histograms of exponentially growing cells (left), and after replication run-out (right) are in black for the wild type cells and in grey for the cells with excess DnaA. 10000 cells were measured and one tick on the ordinate represents 100 cells. (D) The increase in average DNA/mass for the DnaA overproducing cells relative to that of the wild type cells.

Previous work concerning the effect of extra DnaA on the timing of initiation of replication was mainly performed with plasmids containing the *dnaA* gene under control of inducible promoters, presumably leading to quite high DnaA concentrations [33–35,38]. To see the effect of a higher concentration of DnaA, but to avoid complications with a burst of production after induction, cells containing a plasmid (pACYC184) bearing the *dnaA* gene under control of its own promoter were analyzed. These cells had a DnaA concentration that was on average 35, 10 and 11 times higher than the wild type cells when grown in acetate, glucose and GluCAA medium, respectively (Table 1 (MOR90)). We expect that large overproduction of DnaA leads to heterogeneity in growth parameters in the cell population and it has been shown that a large excess of DnaA leads to replication fork collapse [47]. Therefore the conditions for proper cell cycle analysis were not present and accordingly the cell cycle parameters were not simulated for these cells. The DNA contents per cell were found to be higher than normal in the cells with a large excess of DnaA (Fig 2 and S4 Table). For the cells grown in GluCAA medium the increase in DNA/mass was quite low (5%) (Fig 2D and S4 Table). This and the high degree of similarity of the exponential histograms of the wild type and DnaA overproducing cells (Fig 2C) indicated that the amount of over-replication was quite modest. The difference between over-initiation and over-replication may be explained by the disintegration of some replication forks. It has been shown in cells with a large excess of DnaA that some of the replication forks collapse shortly after initiation [47]. Thus, the low increase in the DNA concentration of the exponentially growing cells after a large overproduction of the DnaA might partly be due to such replication fork collapse. For the replication run-out histograms larger differences were found (Fig 2, rightmost panels). The DnaA overproducing cells yielded peaks at higher chromosome equivalents than what would be expected from the corresponding DNA distributions of the exponentially growing cells. It has previously been shown that if cells contain a surplus of DnaA, initiations might sometimes occur during the rifampicin treatment [23,31,34]. Some of the asynchrony observed here is also probably due to such rifampicin resistant initiations.

### Lack of DDAH leads to a change in the timing of replication during slow growth

The above results indicate that when a massive excess of DnaA is present, the otherwise quite robust cell cycle machine breaks down. A similar situation is seen if the RIDA system is inactivated causing massive over-initiation and lethality. We wished to investigate a situation where a less important part of the DnaA activity control, DDAH, was missing. To do so, we investigated cells where the *datA* site had been deleted.

**Fig 3:**
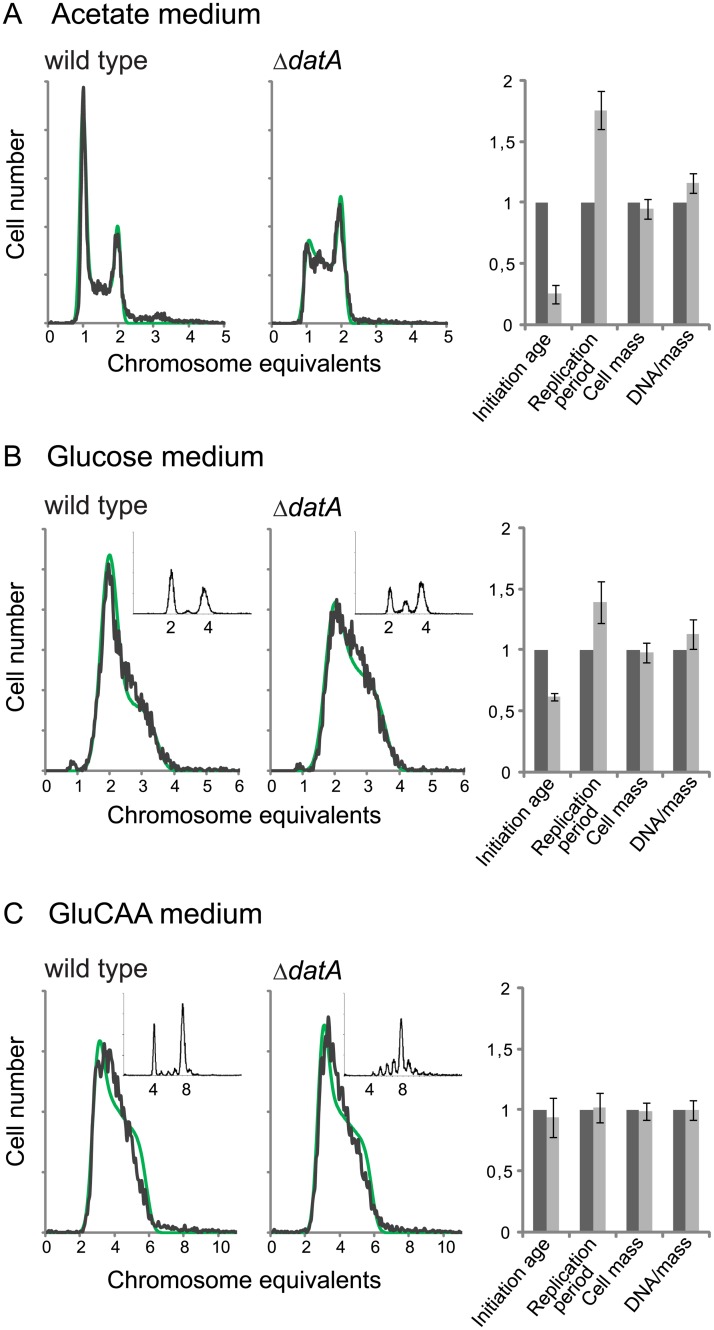
Deletion of the *datA* region changes the cell cycle parameters only during slow growth. DNA histograms of exponentially growing Δ*datA* and wild type cells (MOR177 and MG1655) grown in acetate medium (A), glucose medium (B) and GluCAA medium (C). The run-out DNA histograms are inserted for (B) and (C).). The theoretical curves from the best-fit simulations are shown in green with the experimental DNA histograms in black. See legend to Fig 1 for further details. The calculated values for initiation age, replication period, cell mass and DNA/mass relative to the values for the wild type are shown (A-C, rightmost panel). The values are an average of three or more experiments and the error bars represent the standard deviation. For a more detailed description of the cell cycle parameters see S4 Fig.

We found that a deletion of the *datA* site did not lead to significant changes in the cellular DnaA concentration (Table 1 (MOR177)). When grown in acetate medium, the Δ*datA* cells had about the same doubling time (S5 Table), cell mass and DNA concentration as wild type cells (Fig 3A, rightmost panel and S5 Table). However, the number of cells containing one chromosome was much lower for the Δ*datA* cells compared to the wild type (Fig 3A). This means that the Δ*datA* cells initiate at a lower age. The reduction in initiation age was found to be about 75% and was accompanied by an increase in the length of the replication period compared to the wild type cells (Fig 3A, rightmost panel and S5 Table). The wild type and the Δ*datA* cells had about the same average cell mass. Thus, the initiating Δ*datA* cells must also be smaller than the initiating wild type cells.

Also in cells grown in glucose initiation occurred earlier in the cell cycle in the Δ*datA* cells compared to the wild type cells, but the effect was not as pronounced as in the cells grown in acetate medium (Fig 3B). Also in this case, early initiation was accompanied by an increase in the duration of the replication period (Fig 3B, rightmost panel and S5 Table).

### Lack of DDAH regulation does not affect cell cycle parameters in GluCAA medium

It has previously been shown that the replication pattern and *oriC*/*ter* ratio are not changed by deletion of the *datA* site in cells grown in GluCAA medium [31]. Because these experiments were performed in medium lacking uridine, which affects the replication pattern of MG1655 [51], we investigated this for cells growing in GluCAA medium containing uridine. Also in this medium the DNA histogram of the exponentially growing cells was the same with and without the *datA* site (Fig 3C). The result shows that the cells grown in GluCAA medium initiate replication at the same time in the cell cycle and that the length of the replication period is the same irrespective of whether the *datA* site is present or not (Fig 3C, rightmost panel and S5 Table). However, a difference is seen in the run-out DNA histogram (Fig 3C, small histograms), which shows asynchronous initiations. This phenotype has also been observed previously and represents initiations occurring during rifampicin treatment [31].

### A five-fold excess of DiaA protein is capable of holding back premature and rifampicin resistant initiations of replication in cells that lack DDAH regulation

DiaA is a DnaA interacting protein which has previously been proposed to have both a positive and negative influence on the initiation process [14–16]. To investigate the effect of DiaA with respect to the timing of initiation we studied cells transformed with plasmids carrying the *diaA* gene under control of its own promoter in wild type cells and in combination with a large excess of DnaA and deletion of *datA*. The strains containing the *diaA* plasmid had a DiaA concentration that was about 5 times higher than the wild type in acetate medium and 4 times higher than the wild type in GluCAA medium (Table 4).

**Table 4:** DiaA concentration in DiaA overproducing wild type cells and Δ*datA* cells.

Strain	Medium	Relative DiaA concentration^1)^
IF97	Acetate	5.0 ± 0.94
IF97	GluCAA	4.1 ± 0.79
IF105	Acetate	5.7 ± 1.12
IF105	GluCAA	3.8 ± 0.53

^1)^ ng DiaA per μg cell extract. The numbers are relative to the wild type or Δ*datA* containing the empty plasmid. Measured by imunoblotting.

± represents the standard deviation

**Fig 4:**
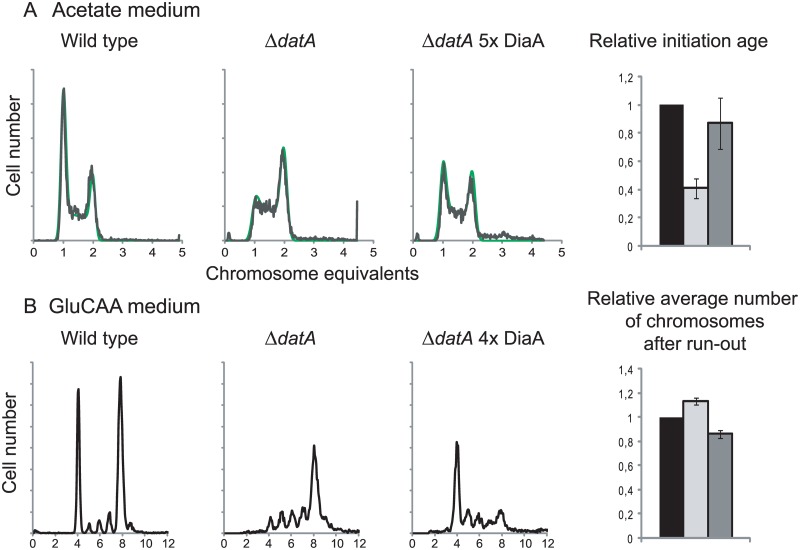
DiaA can hold back premature and rifampicin resistant initiations. Flow cytometry DNA histograms of wild type cells (MG1655 with pACYC184, IF26) (left histogram), Δ*datA* cells with pACYC184 (IF104) (middle histogram) and Δ*datA* cells with pACYC184*diaA* (IF105) (right histogram) grown in acetate medium (A) or GluCAA medium (B). For the cells grown in acetate DNA histograms of exponentially growing cells are shown, while for the cells grown in GluCAA the rifampicin run-out histograms are shown. See legend to Fig 1 for further details. For the cells grown in acetate the initiation ages of the Δ*datA* cells without (light grey bar) or with (dark grey bar) extra DiaA relative to the wild type control (black bar) are shown in the bar histogram in the rightmost panel. The values are an average of three experiments and the error bars represent the standard deviation. For the cells grown in GluCAA the average number of chromosomes for the Δ*datA* cells without (light grey bar) or with (dark grey bar) extra DiaA relative to the wild type control (black bar) are shown in the bar histogram in the rightmost panel. The values are an average of three experiments and the error bars represent the standard deviation.

In wild type cells grown in acetate or GluCAA medium an elevated level of DiaA did not lead to any significant changes in the timing of initiation (S5 Fig). However, in the Δ*datA* cells grown in acetate, which have a lower initiation age compared to wild type cells, the presence of extra DiaA led to a reversal of the phenotype (Fig 4A). In this situation, we observed an increase in the initiation age in the Δ*datA* cells when they in addition expressed extra DiaA (Fig 4A, rightmost panel). This indicates a possible inhibitory role for DiaA. Also in rapidly growing cells, an inhibitory effect of extra DiaA was seen in the Δ*datA* cells. These cells exhibit an asynchrony and over-initiation phenotype as a result of rifampicin-resistant initiations (Fig 3C and 4B) [31]. We observed that the number of rifampicin-resistant initiations was reduced when the Δ*datA* cells also had a 4 times higher concentration of DiaA (Fig 4B). Both results support the idea of an inhibitory role for the DiaA protein at the origin.

It has previously been shown that a *diaA* deletion strain had a somewhat increased concentration of DnaA [14], so we checked whether overproduction of DiaA led to a reduction in the DnaA concentration in the cells. However, we found that the DnaA concentration was not reduced in the cells with an extra supply of DiaA (Table 1 (IF97)).

## Discussion

### DnaA is not the limiting factor for initiation of replication during exponential growth

We show here that extra DnaA does not lead to a shift in the timing of initiation of replication in any of the growth media tested. We found that cells were insensitive to a more than 50% increase in the concentration of ATP-DnaA. Thus, the result indicates that in cells with a substantial surplus of ATP-DnaA, timing of initiation is controlled in the same way as in wild type cells, and that the controller is not the amount of ATP-DnaA. The amount of DnaA in the cell could still be an important parameter that, for instance, helps couple replication rate to growth rate [52], but it does not seem to determine the precise timing of initiation during steady-state growth in the media tested here. The idea that DnaA is not the limiting factor for initiation of replication is also supported by several other lines of evidence. Previously we have shown that the amount of DnaA per origin in the cells varies with the growth medium /growth rate and that there is more DnaA per origin in cells grown in acetate medium compared to cells grown in richer media [23]. It has also been shown that for cells growing slowly with about the same doubling time, the amount of DnaA required per origin at the time of initiation in chemostat-grown cells is considerably lower than the amount required in batch-grown cells [41]. In cells with mini-chromosomes, and therefore many extra origins to initiate, initiation occurs at the same time and mass in the cell cycle [53]. Support for this idea also comes from a recent study showing that the timing of initiation was unchanged in cells with a 20% decrease in the level of DnaA compared to the wild type cells [42]. These and our results indicate that DnaA is not the limiting factor for initiation of replication during steady-state growth.

That the DnaA protein does not regulate timing of initiation is also apparent in *Bacillus subtilis*. *B*. *subtilis* mutant cells which are abnormally small because of an aberrancy in the regulation of cell division were found to initiate replication at the same cell age as wild type cells [54]. This means that these cells initiate replication at a smaller size, with less DnaA protein available, compared to the wild type cells, which again means that the wild type cells were not limited by the amount of DnaA. The result instead implies that there was sufficient DnaA for initiation to occur and that a different signal must have decided when initiation occurred. Interestingly, small *E*. *coli* cells with a similar aberrancy in regulation of cell division [55] were found to behave differently and did not keep the replication pattern irrespective of cell size, but instead kept the cell size at initiation and not the replication pattern [54]. In other words, initiation of replication occurred in older cells in the abnormally small *E*. *coli* cells. The authors induced a transient increase in the amount of DnaA and found a change after run-out of replication. This result might be taken to indicate that cells must accumulate a certain amount of DnaA before initiation could occur. However, with a transient induction, i.e. an unbalanced situation, it is difficult to interpret how the regulation works. We find it likely that the regulatory circuits in *B*. *subtilis* and *E*. *coli* working on the initiation machinery simply are different, and that in neither case does the point in the cell cycle of reaching enough ATP-DnaA decide the time of initiation.

Taken together, the results indicate that although the DnaA protein is highly conserved through evolution both as an initiator and a transcription factor [53] the precise regulatory circuits that govern its activity may not be conserved. This is perhaps not surprising since *B*. *subtilis* and *E*. *coli* are very distantly related.

### A massive excess of ATP-DnaA causes a breakdown of the cell cycle machine

Our results do not fit with the current model for regulation, which assumes that initiation occurs as soon as a certain amount of active DnaA is available [9,56]. This model is based on previous findings where overproduction of the DnaA protein was reported to lead to over-initiation and it was therefore concluded that DnaA was the limiting factor for initiation of replication [33–38]. However, many of these experiments were performed with quite high copy-number plasmids carrying the *dnaA* gene under control of different inducible promoters. This probably led to levels of DnaA that were quite high compared to the level in wild type cells. Also in our work we found that over-initiation occurred when a large surplus of DnaA was present. Thus, it seems clear both from our results and from previous studies, that a large excess of DnaA is capable of initiating origins that would not otherwise initiate. However, these initiations represent a break-down of the cell cycle machine. They are unregulated and probably represent a forced reaction, which is due to the large excess of DnaA protein. Also, the over-initiation appears to be higher than the over-replication because the increase in the DNA/mass was found to be quite modest, especially in the rapidly growing cells (10%). This is also the case in many of the previous studies where the observed increase was only up to about 20% [33,35,38,39]. It has been shown that many of the replication forks collapse in cells with a large excess of DnaA [47]. Thus, the low increase in the DNA concentration after a large overproduction of the DnaA might be due to replication fork collapse. Also, overproduction of DnaA has previously been reported to lead to initiations occurring during the incubation with rifampicin [31,34], a fact that was not known in some of the earlier studies. Therefore, an increase in the number of origins per cell was in many cases interpreted as over-initiation [35,37,38], when it in fact could be a result of rifampicin-resistant initiations.

Our results are not in accordance with one earlier study in which a substantial increase in the DNA/mass was seen after moderate overproduction of DnaA [34]. The reason for this discrepancy is not known, but one difference between our work and this work is that it was *Salmonella typhimurium* and not *Escherichia coli* DnaA protein that was expressed. Another difference is that in the previous work the extra DnaA was expressed form an IPTG inducible promoter which necessarily leads to transient changes in the DnaA level rather than a steady state expression as in our case.

### Removal of the DDAH regulation affects the cell cycle machinery only during slow growth

Previously it has been shown that during rapid growth in GluCAA medium Δ*datA* cells exhibit rifampicin resistant initiations, i.e. initiations occur during the incubation with rifampicin. No changes are seen in the cell cycle of exponentially growing cells [31]. Our work confirms this result, but also shows that removal of the *datA* site in cells growing more slowly in glucose or acetate medium has a different effect compared to in the rapidly growing cells. Under these growth conditions, a change in the cell cycle was seen also in the exponentially growing cells; the Δ*datA* cells initiated earlier in the cell cycle (as smaller cells) compared to the wild type cells. These results demonstrate that the regulatory influence of DDAH on the cell cycle machinery changes with the growth conditions.

The DDAH system (the binding of DnaA and IHF to *datA*) causes conversion of ATP-DnaA to ADP-DnaA [27]. This means that the cell has two systems for inactivating the active form of the DnaA protein; the RIDA (Regulatory Inactivation of DnaA) system and the more recently discovered DDAH system. In our cells with two-fold extra DnaA the RIDA and DDAH systems ensure that about 60% of the total DnaA is in the ADP-form so that although there is an elevated level of ATP-DnaA compared to the wild type cells there is still a balance in the cell between the two forms of the DnaA protein. Mutations which block the RIDA system are lethal due to over-initiation [29,30]. This shows that the cell cycle machinery is dependent on conversion of ATP-DnaA to ADP-DnaA in order to work. That the cell cycle can work when *datA* is deleted (i.e. without DDAH) indicates that RIDA is the more important of the two systems.

A difference between the DDAH and RIDA systems is that the RIDA system is dependent on ongoing replication whereas DDAH is not [57]. In rapidly growing cells with overlapping replication cycles there are always active replication forks in the cell which means that the RIDA system will always be active. Thus, the DDAH system may be of less importance in taking down the level of ATP-form DnaA in rapidly growing compared to slowly growing cells. In accordance with this assumption, major changes in the level of ATP-DnaA after deletion of the *datA* site was not found in rapidly growing cells [58]. However, in the acetate grown cells only around 25% of the cells contain active replication forks while the rest of the cells in the population are either in the B- or the D-period where no replication occurs. It might therefore be that the DnaA-inactivating activity of DDAH becomes more important under such conditions. It is possible that loss of DDAH in slowly growing cells leads to a shift in the balance between the ATP-form and ADP-form of DnaA that is larger than what we get with two-fold extra DnaA (where still a lot of this is in the ADP-form). This unbalance in the ratio of ATP-DnaA and ADP-DnaA could lead to premature initiations similar to the situation with a large surplus of DnaA.

### Excess DiaA has an effect only in cells with compromised cell cycles

The DiaA protein has been shown to affect the initiation process both positively and negatively *in vitro* [14–16], and it has been suggested that DiaA might have a dual role in the initiation process. First, a stimulatory role early in the initiation process where it aids in the recruitment of the DnaA to the origin and in formation of the open complex, and later an inhibitory role where it inhibits too early loading of the DnaB helicase and the rest of the replication machinery [16]. How the transition between the DiaA-bound inactive complex and the DnaB-bound active replicative complex might occur is not known. We show in this work that over-production of DiaA does not have any effect in an otherwise wild type situation. This result indicates that the transition to the DnaB-bound active replicative complex is not simply a question of a competition between DiaA and DnaB for binding to DnaA, as one would then expect initiation to be delayed when a surplus of DiaA is present. No such delay was observed. It might therefore be an active mechanism or a signal that leads to release of DiaA from its binding site, allowing DnaB to bind.

In contrast to the wild type cells, we did see an effect of extra DiaA in cells lacking the DDAH system where the too early and rifampicin resistant initiations were reduced by expression of extra DiaA. This indicates an inhibitory role for DiaA under these circumstances and shows that in cells with a less robust or a compromised cell cycle additional changes in the levels of regulators/components of the cell cycle, such as DiaA, is more likely to have an effect. These results support the previously suggested inhibitory role for the DiaA protein [16] and a possible explanation of the results is that the extra DiaA in the Δ*datA* cells inhibits premature and rifampicin resistant initiations by inhibiting the loading of the DnaB helicase.

### The regulation of initiation of replication is complex and varies with the growth conditions

Regulation of initiation must fulfill two requirements. It must prevent extra initiation events, and it must ensure sufficient initiation so that one initiation event occurs per generation per origin. Several mechanisms have been discovered that ensure that extra initiation events do not occur (origin sequestration, inactivation of DnaA, inhibition of *dnaA* transcription), but less is known about the timing of replication initiation, i.e. the rate limiting steps, and whether the same factor(s) are required under all conditions. The frequency of replication must match the growth rate, otherwise the cellular DNA concentration will be altered. We propose that i) *E*. *coli* has a robust replication cycle driven by the cycling of the levels of ATP-DnaA and ADP-DnaA, ii) initiation cannot occur unless sufficient amounts of ATP-DnaA at *oriC* has licenced this event and iii) signals depending on the cell’s environment govern the exact timing of initiation.

## Materials and Methods

### Bacterial strains, plasmids and growth conditions

All strains used are *Escherichia coli* K-12 and are listed in Table 5. Cells were grown in AB minimal medium [59] supplemented with 10 μg/ml thiamine, 25 μg/ml uridine and either 0.4% sodium acetate, 0.4% glucose or 0.4% glucose and 0.5% casamino acids at 30°C (Acetate and glucose medium) or 37°C (GluCAA medium). For determination of the ATP-DnaA to ADP-DnaA ratio, cells were grown in low phosphate medium (see below). The growth rates were determined by measuring the optical density of the cultures at 450 nm.

**Table 5:** Strains and plasmids.

Strain	Relevant genotype	Reference
MG1655	Wild type	[67,68]
IF72	MG1655 *attλ*::pAH150_*dnaA*	This work
MOR177	MG1655 Δ*datA*	[31]
IF26	MG1655/pACYC184	This work
MOR90	MG1655/pACYC184_*dnaA*	[61]
IF97	MG1655/pACYC184_*diaA*	This work
IF104	MOR177/pACYC184	This work
IF105	MOR177/pACYC184_*diaA*	This work
KS1411	MG1655/pFH2102	This work
KS1412	MG1655/pFH2102_*dnaN*	This work
KS1413	IF72/pFH2102	This work
KS1414	IF72/pFH2102_*dnaN*	This work

IF72 was made by amplification of the *dnaA* gene including the promoter region with the primers 5`-CGAGGATCCTTACGATGACAATGTTCTG and 5`-CGGAGCTCGGCTTTATTGGATATCCG. This fragment was ligated into the vector pAH150 and the resulting plasmid pAH150_*dnaA* was integrated into the chromosome at the *λ att* site as described in [60]. After construction both copies of the *dnaA* gene in IF72 were sequenced to ensure that no mutation had been introduced.

The pACYC184_*dnaA* plasmid was constructed in previous work [61]. The plasmid pACYC184_*diaA* was made by amplifying the *diaA* gene with the upstream promoter region (derived from [14]) with the primers 5`GCACTGCAGGTTAACCACCAAACAGAC and 5`CGAGGATCCTTAATCATCCTGGTGAGG followed by sub-cloning in the pGEM-T-Easy vector (Promega) and ligation of the gene fragment into the *EcoRI* site of pACYC184.

The pFH2102_*dnaN* plasmid was made by amplifying the *dnaN* gene with the primers 5`GGCGGATCCATGAAATTTACCGTAGAAGCTGAG and 5`GGCGAATTCTTACAGTCTCATTGGCATGACAAC and ligation of the gene fragment into the *BamHI* and *EcoRI* sites of pFH2102. The two plasmids were then transformed into MG1655 and IF72 by electroporation.

### Flow cytometry analysis and calculation of cell cycle parameters

Exponentially growing cells (OD ~ 0.15) were harvested and fixed in 70% ethanol or treated with 300 μg ml^-1^ rifampicin and 10 μg ml^-1^ cephalexin for two or more generations before fixation to inhibit new rounds of initiation and cell division, respectively [46]. Flow cytometry was performed with a LSR II flow cytometer (BD Biosciences). Total protein content in the cells was stained with Fluorescein isothiocyanate (FITC, Sigma-Aldrich) and used to calculate the average mass [62]. The DNA was stained with Hoechst 33258 (Sigma-Aldrich) [41]

The cell cycle parameters (initiation age, replication period) were calculated by combining the data from the flow cytometry analysis, the theoretical age distribution of an exponential culture and the generation time obtained by OD measurements in an excel based simulation program [44]. For cells that have only one round of ongoing replication (cells grown slowly in acetate), the simulation program also provides the percentage of cells found in the respective periods of the cell cycle, i.e. in B-, C- and D-period.

The length of the replication period (C-period) was also calculated with an independent method using the *oriC*/*ter* ratios obtained by quantitative PCR and the doubling time (τ) with the formula *oriC*/*ter* = 2^C/τ^ (See Supporting information).

Whereas one example histogram is shown in each panel of the figures, the values of cell cycle parameters are the average calculated for samples from several experiments.

### Quantification of DnaA, DnaN and DiaA

Exponentially growing cells (OD ~ 0.15) were harvested by centrifugation and SDS (sodium-dodecyl-sulfate) samples of cell extracts and purified proteins were prepared as previously described [63]. Samples were subjected to 12% SDS-polyacrylamide gel electrophoresis and detection of DnaA was carried out using anti-DnaA-antibody, anti-DnaN-antibody or anti-DiaA-antibody and ECF fluorescence kit (GE Healthcare). Quantification was performed using Image Quant software (Molecular Dynamics).

### RNA isolation and sequencing

RNA was isolated using SV Total RNA isolation system (Promega) from MG1655 and IF72 cells grown exponentially (OD ~0.15) in GluCAA medium. The isolated RNA was analyzed on an Agilent Bioanalyzer to confirm quality and depleted for rRNA using Ribo-Zero rRNA Removal Kit for Gram-Negative Bacteria (Illumina).

The rRNA depleted samples were submitted to the Norwegian Sequencing Centre (sequencing.uio.no) where libraries were prepared using TruSeq stranded mRNA reagents (Illumina) according to manufacturer’s instructions, entering the procedure at the RNA fragmentation step with 35 ng rRNA-depleted RNA, and fragmenting for 4 minutes at 94°C. Libraries were sequenced on an Illumina NextSeq-500 instrument with 150 cycle mid-output v1 reagents, according to manufacturer's instructions, employing 75 bp paired-end reads. Image analysis and base calling were performed using Illumina's RTA software version 2.1.3. Reads were filtered to remove those with low base call quality using Illumina's default chastity criteria.

The sequencing was performed on three replicates from each of the two strains.

### RNA sequence analysis

The sequence reads were received from the Norwegian Sequencing Centre as FASTQ files containing the forward and reverse reads respectively and were analyzed using the tool Rockhopper [64]. The expression level of each transcript is reported using RPKM (Reads Per Kilobase per Million mapped reads), except that instead of dividing by the total number of reads it is divided by the upper quartile of gene expression. To test for differentially expressed genes the software first uses local regression to obtain a smooth estimate of gene expression variances. Then, for each transcript, a statistical test for the null hypothesis, which is that the expression of the transcript is the same in different conditions, is performed. The Negative Binomial distribution is used as the statistical model to compute a p-value indicating the probability of observing a transcript's expression levels in different conditions by chance. Because multiple test are being performed, q-values are reported that control the false discovery rate using the Benjamini-Hochberg procedure [64]. Genes with a *q*-value ≤0.01 were considered as significantly differentially expressed. To verify differentially expressed genes RT-QPCR was performed (see Supporting information, S8 Table and S9 Table).

### Determination of cellular levels of the adenine nucleotide forms of DnaA protein

Cells grown in TG640-thy-less medium with 0.2% glucose and 40 μg/ml of each amino acid overnight were diluted to OD_660_ = 0.005 in TG320-thy-less medium and 0.28 mCi of HPO_4_^2-^ (^32^P) was added to 1 ml of cells (3x1 ml). Subsequently, cells were grown exponentially at 37°C to OD_660_ = 0.20 and harvested. Cell extracts were made and immuno-precipitated with purified DnaA antiserum as described [65]. To remove unspecific immunoglobulins from the DnaA rabbit antiserum (R22) it was purified by passage through a column containing all cellular proteins except DnaA. The column was made of cyanogen bromide activated Sepharose beads coupled to extract from a *dnaA* deletion strain.

After immuno-precipitation the nucleotides bound to DnaA (ATP or ADP) were extracted and separated on thin layer chromatography (TLC) in 1M HCOOH containing 0.8M LiCl. Migration of ATP and ADP was determined by mobility of cold ATP and ADP. The amounts of ATP and ADP were quantified in ImageQuant, taking into consideration the different numbers of phosphate groups in ATP and ADP. The percentage ATP from each sample was then calculated.

## Supporting Information

S1 Fig Determination of DnaA concentrations by immunoblotting. To quantify the concentration of DnaA, exponentially growing cells (OD ~ 0.15) were harvested, treated with SDS and the indicated amount of cell extract was subjected to 12% SDS-polyacrylamide gel electrophoresis. Detection of DnaA was carried out using anti-DnaA-antibody and ECF fluorescence kit (GE Healthcare). Quantification was performed using Image Quant software (Molecular Dynamics). The experiments were repeated 3 times or more and the average numbers are given in Table 1.
(PDF)

S2 Fig Calculated cell cycle parameters for wild type and cells with a two-fold increase in the DnaA concentration. A linear representation of the length of the different cell cycle periods for the wild type and the cells with two-fold extra DnaA grown in minimal medium supplemented with acetate (A), glucose (B) or GluCAA (C). For slowly growing cells like the cells grown in acetate, which do not have overlapping rounds of replication, the time from the cell is newborn until it initiates a new round of replication is called the B period and represents the time where no replication is occurring. Here this is drawn as a grey line. For the more rapidly growing cells where initiation occurs in one of the previous generations, the previous round of replication is not yet finished in the newborn cell. Thus, these cells do not have a B-period. Instead the initiation age (a_i_), the time point where the cells initiate a new round of initiation is indicated. The time the cells use to replicate the chromosome is called the C-period (replication period) and is represented by the red line. Finally, the time between the end of replication and division is called the D-period and is represented by the black line. The arrow represents a time axis with the average doubling time of the respective strain indicated. Each line indicates one generation and the number of lines indicates the generations spanned by C + D. The calculated values are an average of three or more experiments and the standard deviations are given in S1 Table.
(PDF)

S3 Fig DNA histograms and calculated cell cycle parameters for wild type cells with a two-fold increase in the DnaA concentration grown in low phosphate medium. To measure the amount of ATP and ADP-DnaA in the cells the cells have to be grown in a low-phosphate medium. We also analyzed cells grown in this medium with flow cytometry and calculated the cell cycle parameters. DNA histograms of the wild type and the cells with two-fold extra DnaA is shown to the left. The black lines represent the experimental values and the green line the theoretical simulation. Replication run out histograms are shown as insets. To the right a linear representation of the length of the different cell cycle periods for the wild type and the cells with two-fold extra DnaA is shown. The calculated values are an average of three experiments. No significant difference was found between the wild type cells and the cells with two-fold extra DnaA.
(PDF)

S4 Fig Calculated cell cycle parameters for wild type and Δ*datA* cells. A linear representation of the length of the different cell cycle periods for the wild type and the Δ*datA* cells grown in medium supplemented with acetate (A), glucose (B) or GluCAA (C). See legend to S1 Fig for further details. The calculated values are an average of three or more experiments and the standard deviations are given in S5 Table.
(PDF)

S5 Fig Excess DiaA has no effect in wild type cells. Flow cytometry DNA histograms of wild type cells and cells with extra DiaA grown in minimal medium supplemented with acetate (30°C) (top panels) and GluCAA (37°C) (bottom panels). Small panels show rifampicin/cephalexin treated cells. The chromosome equivalents are shown on the abscissa and the number of cells on the ordinate. 10000 cells were measured and one tick on the ordinate represents 100 cells. The black curves represent the experimental histograms and the green curves represent the theoretical simulations. Average values of the cell cycle parameters from simulations of three or more experiments are shown as linear representations to the left of the histograms. Each line indicates one generation and the number of lines indicates the generations spanned by C + D.
(PDF)

S1 Table Cell cycle parameters of wild type cells and cells with two-fold extra DnaA. (PDF)

S2 Table Determination of DnaN concentrations in MG1655 and IF72 by immunoblotting. (PDF)

S3 Table Cell cycle parameters of wild type cells and cells with extra DnaN. (PDF)

S4 Table Average mass and DNA content of wild type cells and cells with large excess DnaA. (PDF)

S5 Table Cell cycle parameters of wild type cells and Δ*datA* cells. (PDF)

S6 Table Replication periods determined by QPCR. To obtain *oriC*/*ter* ratio using quantitative PCR, chromosomal DNA was purified from exponential GluCAA cultures (OD = 0.15). Quantitative PCR performed as described in [66] and normalized to a sample where the *oriC*/*ter* ratio is 1:1. The replication period was calculated from the *oriC*/*ter* ratio and the doubling time (τ) (S1 Table–S3 Table) using the formula *oriC*/*ter* = 2^C/τ^. For cells grown in acetate and glucose the differences in the *oriC/ter* ratios are too small to get reliable results with quantitative PCR.
(PDF)

S7 Table Complete list of transcripts obtained by RNA sequencing. (XLSX)

S8 Table Verification of differentially expressed genes by RT-qPCR. cDNA was generated using SuperScript III Reverse Transcriptase (Life Technologies) according to the manufacturers protocol using random hexamer primers (Life Technologies). Quantitative PCR was performed as described in [66] with specific primers (listed in S9 Table). The relative mRNA levels were normalized to the housekeeping gene rrsA encoding 16S rRNA in *E*.*coli*.
(PDF)

S9 Table Primers used for RT-qPCR. (PDF)
