# Supplementary material for: The DnaA Protein Is Not the Limiting Factor for Initiation of Replication in Escherichia coli
Source: PLoS Genet. 2015 Jun 5;11(6):e1005276. doi: 10.1371/journal.pgen.1005276 (PMC4457925; doi:10.1371/journal.pgen.1005276)

Figure S1: Determination of DnaA concentrations by immunoblotting

IF72 compared to wild type cells

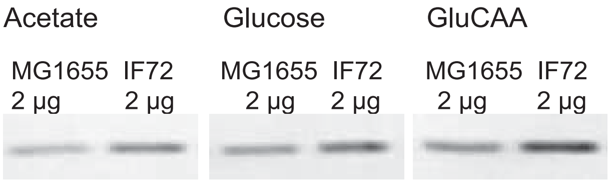

MOR90 compared to wild type cells with empty vector

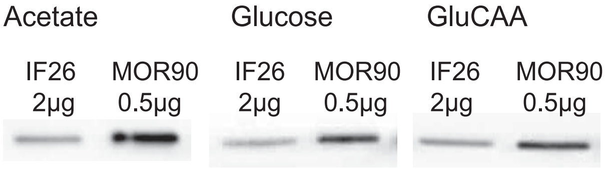

Supplement: S1 Fig — To quantify the concentration of DnaA, exponentially growing cells (OD ~ 0.15) were harvested, treated with SDS and the indicated amount of cell extract was subjected to 12% SDS-polyacrylamide gel electrophoresis. Detection of DnaA was carried out using anti-DnaA-antibody and ECF fluorescence kit (GE Healthcare). Quantification was performed using Image Quant software (Molecular Dynamics). The experiments were repeated 3 times or more and the average numbers are given in Table 1. (PDF) [file pgen.1005276.s001.pdf]
