# Supplementary material for: The DnaA Protein Is Not the Limiting Factor for Initiation of Replication in Escherichia coli
Source: PLoS Genet. 2015 Jun 5;11(6):e1005276. doi: 10.1371/journal.pgen.1005276 (PMC4457925; doi:10.1371/journal.pgen.1005276)

Figure S2: Calculated cell cycle parameters for wild type and cells with a two fold increase in the DnaA concentration

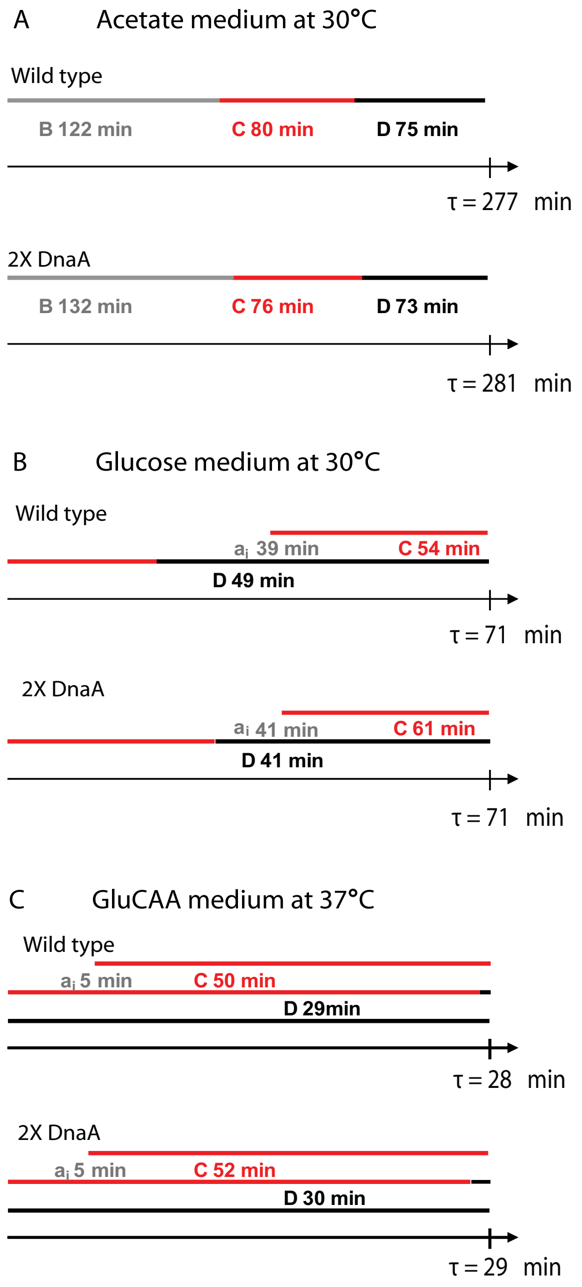

Supplement: S2 Fig — A linear representation of the length of the different cell cycle periods for the wild type and the cells with two-fold extra DnaA grown in minimal medium supplemented with acetate (A), glucose (B) or GluCAA (C). For slowly growing cells like the cells grown in acetate, which do not have overlapping rounds of replication, the time from the cell is newborn until it initiates a new round of replication is called the B period and represents the time where no replication is occurring. Here this is drawn as a grey line. For the more rapidly growing cells where initiation occurs in one of the previous generations, the previous round of replication is not yet finished in the newborn cell. Thus, these cells do not have a B-period. Instead the initiation age (ai), the time point where the cells initiate a new round of initiation is indicated. The time the cells use to replicate the chromosome is called the C-period (replication period) and is represented by the red line. Finally, the time between the end of replication and division is called the D-period and is represented by the black line. The arrow represents a time axis with the average doubling time of the respective strain indicated. Each line indicates one generation and the number of lines indicates the generations spanned by C + D. The calculated values are an average of three or more experiments and the standard deviations are given in S1 Table. (PDF) [file pgen.1005276.s002.pdf]
