# Supplementary material for: The DnaA Protein Is Not the Limiting Factor for Initiation of Replication in Escherichia coli
Source: PLoS Genet. 2015 Jun 5;11(6):e1005276. doi: 10.1371/journal.pgen.1005276 (PMC4457925; doi:10.1371/journal.pgen.1005276)

Figure S3: Calculated cell cycle parameters for wild type cells with a two fold increase in the DnaA concentration in low phosphate medium

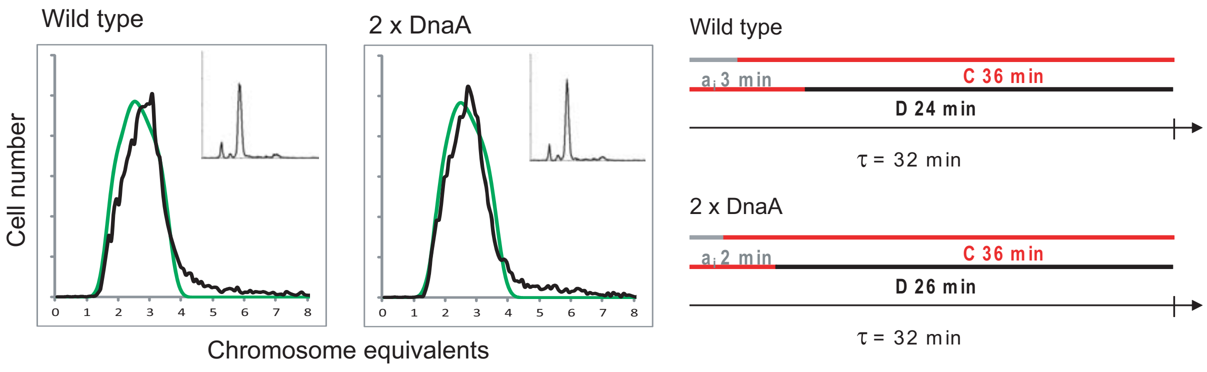

Supplement: S3 Fig — To measure the amount of ATP and ADP-DnaA in the cells the cells have to be grown in a low-phosphate medium. We also analyzed cells grown in this medium with flow cytometry and calculated the cell cycle parameters. DNA histograms of the wild type and the cells with two-fold extra DnaA is shown to the left. The black lines represent the experimental values and the green line the theoretical simulation. Replication run out histograms are shown as insets. To the right a linear representation of the length of the different cell cycle periods for the wild type and the cells with two-fold extra DnaA is shown. The calculated values are an average of three experiments. No significant difference was found between the wild type cells and the cells with two-fold extra DnaA. (PDF) [file pgen.1005276.s003.pdf]
