# Supplementary figures and images for: The DnaA Protein Is Not the Limiting Factor for Initiation of Replication in Escherichia coli
Source: PLoS Genet. 2015 Jun 5;11(6):e1005276. doi: 10.1371/journal.pgen.1005276 (PMC4457925; doi:10.1371/journal.pgen.1005276)

**Figure S4: Calculated cell cycle parameters for wild type and  $\Delta datA$  cells**

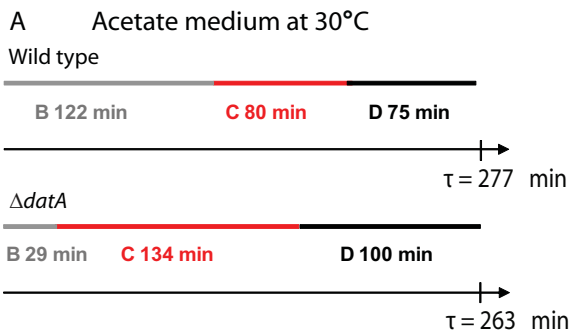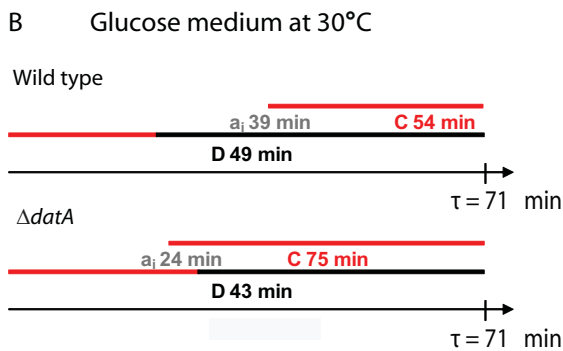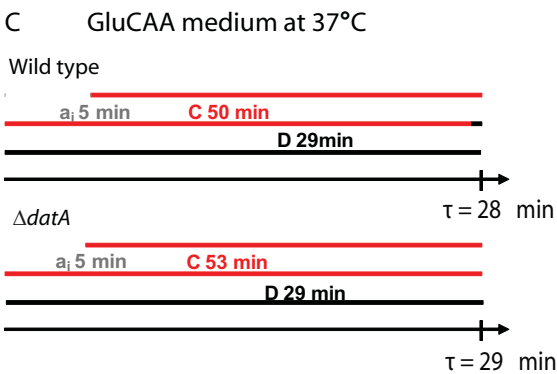

Supplement: S4 Fig — A linear representation of the length of the different cell cycle periods for the wild type and the ΔdatA cells grown in medium supplemented with acetate (A), glucose (B) or GluCAA (C). See legend to S1 Fig for further details. The calculated values are an average of three or more experiments and the standard deviations are given in S5 Table. (PDF) [file pgen.1005276.s004.pdf]

**Figure S5: Excess DiaA has no effect in wild type cells**

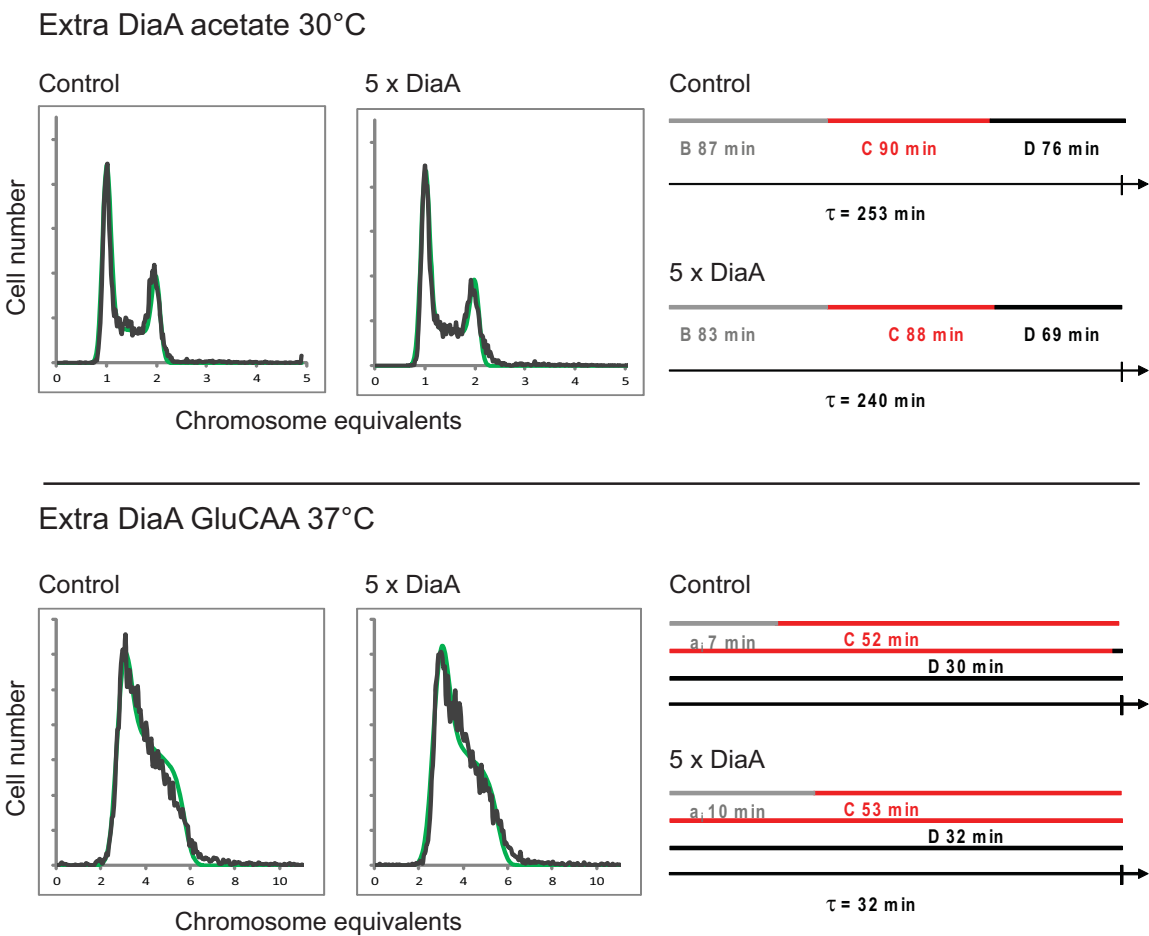

Supplement: S5 Fig — Flow cytometry DNA histograms of wild type cells and cells with extra DiaA grown in minimal medium supplemented with acetate (30°C) (top panels) and GluCAA (37°C) (bottom panels). Small panels show rifampicin/cephalexin treated cells. The chromosome equivalents are shown on the abscissa and the number of cells on the ordinate. 10000 cells were measured and one tick on the ordinate represents 100 cells. The black curves represent the experimental histograms and the green curves represent the theoretical simulations. Average values of the cell cycle parameters from simulations of three or more experiments are shown as linear representations to the left of the histograms. Each line indicates one generation and the number of lines indicates the generations spanned by C + D. (PDF) [file pgen.1005276.s005.pdf]
