# Supplementary material for: The DnaA Protein Is Not the Limiting Factor for Initiation of Replication in Escherichia coli
Source: PLoS Genet. 2015 Jun 5;11(6):e1005276. doi: 10.1371/journal.pgen.1005276 (PMC4457925; doi:10.1371/journal.pgen.1005276)
Supplement: S1 Table — (PDF) [file pgen.1005276.s006.pdf]

Table S1: Cell cycle parameters of wild type cells and cells with two-fold extra DnaA

	Medium	Doubling time (min)[1)]	Mass[2)]	DNA/ mass[2)]	Initiation age (min)	Initiation age/ doubling time	C-period (min)	C-period/ doubling time
Wild type	Acetate	277 ± 38	1	1	122 ± 10	0.44 ± 0.08	80 ± 16	0.29 ± 0.07
2X DnaA	Acetate	281 ± 41	0.99 ± 0.05	1.06 ± 0.04	132 ± 15	0.47 ± 0.08	76 ± 18	0.27 ± 0.1
Wild type	Glucose	71 ± 3	1	1	39 ± 5	0.55 ± 0.06	54 ± 3	0.76 ± 0.04
2X DnaA	Glucose	71 ± 3	0.92 ± 0.10	1.13 ± 0.15	41 ± 2	0.57 ± 0.01	61 ± 1	0.86 ±.01
Wild type	GluCAA	28 ± 1	1	1	5 ± 1	0.18 ± 0.02	50 ± 5	1.79 ± 0.11
2X DnaA	GluCAA	29 ± 1	0.96 ± 0.07	1.02 ± 0.03	5 ± 1	0.17 ± 0.04	52 ± 6	1.79 ± 0.16

[1)] The experiments were repeated three times or more and were performed at 30°C for cells grown in acetate or glucose medium and at 37°C for cells grown in GluCAA medium. Experiments performed at 37°C for cells grown in acetate or glucose medium and at 30°C for cells grown in GluCAA medium confirmed that cells with 2X DnaA showed a change in cell cycle parameters compared to wild type cells.
[2)] Average values. Relative to the wild type.
± represents the standard deviation
