# Supplementary material for: The DnaA Protein Is Not the Limiting Factor for Initiation of Replication in Escherichia coli
Source: PLoS Genet. 2015 Jun 5;11(6):e1005276. doi: 10.1371/journal.pgen.1005276 (PMC4457925; doi:10.1371/journal.pgen.1005276)
Supplement: S2 Table — (PDF) [file pgen.1005276.s007.pdf]

Table S2: Determination of DnaN concentrations by immunoblotting

**Strain**	**Genotype**	**Relative DnaN concentration[1)]**
MG1655	Wild type	1
IF72	MG1655 *attλ::*pAH150_*dnaA*	1.02 ± 0.22

[1)] The numbers are relative to the wild type. The numbers are an average of three experiments and ± represents the standard deviation.
