# Supplementary material for: The DnaA Protein Is Not the Limiting Factor for Initiation of Replication in Escherichia coli
Source: PLoS Genet. 2015 Jun 5;11(6):e1005276. doi: 10.1371/journal.pgen.1005276 (PMC4457925; doi:10.1371/journal.pgen.1005276)
Supplement: S3 Table — (PDF) [file pgen.1005276.s008.pdf]

Table S3: Cell cycle parameters of wild type cells and cells with extra DnaN grown in GluCAA medium

**Strain**	**Plasmid**	**Relative DnaN concentration[1)]**	**Doubling time (min)**	**Initiation age/ doubling time**	**C-period/ doubling time**
KS1411	MG1655/pFH2102	1	28	0.25 ± 0.04	1.63 ± 0.06
KS1412	MG1655/pFH2102_dnaN	1.36 ± 0.41	26	0.29 ± 0.05	1.60 ± 0.11
KS1413	IF72/pFH2102	1	28	0.22 ± 0.03	1.65 ± 0.04
KS1414	IF72/pFH2102_dnaN	1.85 ± 0.71	26	0.28 ± 0.03	1.59 ± 0.05

[1)] Found by immunoblotting. The numbers are relative to the wild type
The numbers are an average of four experiments and ± represents the standard deviation.
