# Supplementary material for: The DnaA Protein Is Not the Limiting Factor for Initiation of Replication in Escherichia coli
Source: PLoS Genet. 2015 Jun 5;11(6):e1005276. doi: 10.1371/journal.pgen.1005276 (PMC4457925; doi:10.1371/journal.pgen.1005276)
Supplement: S4 Table — (PDF) [file pgen.1005276.s009.pdf]

Table S4: Average mass and DNA content of wild type cells and cells with large excess DnaA

	Medium	Doubling time (min) [1)]	Mass[2)]	DNA content[2)]	DNA/ mass[2)]
Wild type	Acetate	277 ± 38	1	1	1
35X DnaA	Acetate	267	0.80	1.36	1.70
Wild type	Glucose	71 ± 3	1	1	1
10X DnaA	Glucose	86	0.91	1.32	1.45
Wild type	GluCAA	28 ± 1	1	1	1
11X DnaA	GluCAA	32	1.05	1.11	1.06

[1)]Doubling times were obtained at 30°C for cells grown in acetate or glucose medium and at 37°C for cells grown in GluCAA medium
[2)]Average values. Relative to the wild type.
± represents the standard deviation
