# Supplementary material for: The DnaA Protein Is Not the Limiting Factor for Initiation of Replication in Escherichia coli
Source: PLoS Genet. 2015 Jun 5;11(6):e1005276. doi: 10.1371/journal.pgen.1005276 (PMC4457925; doi:10.1371/journal.pgen.1005276)
Supplement: S5 Table — (PDF) [file pgen.1005276.s010.pdf]

Table S5: Cell cycle parameters of wild type cells and Δ*datA* cells

	Medium	Doubling time (min) [1)]	Mass[2)]	DNA/ mass[2)]	Initiation age (min)	Initiation age/ doubling time	C-period (min)	C-period/ doubling time
Wild type	Acetate	277 ± 38	1	1	122 ± 10	0.44 ± 0.08	80 ± 16	0.29 ± 0.07
Δ*datA*	Acetate	263 ± 31	0.95 ± 0.08	1.16 ± 0.08	29 ±10	0.11 ±0.05	134 ± 5	0.51 ± 0.08
Wild type	Glucose	71 ± 3	1	1	39 ± 5	0.55 ± 0.06	54 ± 3	0.76 ± 0.04
Δ*datA*	Glucose	71 ± 3	0.98 ± 0.08	1.13 ± 0.12	24 ±3	0.34 ± 0.04	75 ± 5	1.06 ± 0.11
Wild type	GluCAA	28 ± 1	1	1	5 ± 1	0.18 ± 0.02	50 ± 5	1.79 ± 0.11
Δ*datA*	GluCAA	29 ± 2	0.99 ± 0.07	1.00 ± 0.08	5 ± 2	0.17 ± 0.05	53 ± 2	1.83 ± 0.18

[1)]Doubling times were obtained at 30°C for cells grown in acetate or glucose medium and at 37°C for cells grown in GluCAA medium
[2)]Average values. Relative to the wild type.
± represents the standard deviation
