# Supplementary material for: The DnaA Protein Is Not the Limiting Factor for Initiation of Replication in Escherichia coli
Source: PLoS Genet. 2015 Jun 5;11(6):e1005276. doi: 10.1371/journal.pgen.1005276 (PMC4457925; doi:10.1371/journal.pgen.1005276)
Supplement: S6 Table — To obtain oriC/ter ratio using quantitative PCR, chromosomal DNA was purified from exponential GluCAA cultures (OD = 0.15). Quantitative PCR performed as described in [66] and normalized to a sample where the oriC/ter ratio is 1:1. The replication period was calculated from the oriC/ter ratio and the doubling time (τ) (S1 Table–S3 Table) using the formula oriC/ter = 2C/τ. For cells grown in acetate and glucose the differences in the oriC/ter ratios are too small to get reliable results with quantitative PCR. (PDF) [file pgen.1005276.s011.pdf]

Table S6: Replication periods determined by QPCR

**Strain**	***oriC/ter* ratio**	**Calculated replication period**
Wild type	3.72 ± 0.25	53 ± 3 min
Low excess DnaA	3.77± 0.17	55 ± 1 min
Δ*datA*	3.91± 0.09	55 ± 1 min
Wild type (with empty vector)	3.93[1] (3.51-4.34)	57[1]
High excess DnaA	4.40[1] (4.15-4.64)	67[1, 2]

± represents the standard deviation.

[1]Average of two experiments.

[2] It is not known whether the replication period actually is longer in cells with ten-fold extra DnaA or whether this number is high because some cells suffer replication fork collapse
