# Supplementary material for: The DnaA Protein Is Not the Limiting Factor for Initiation of Replication in Escherichia coli
Source: PLoS Genet. 2015 Jun 5;11(6):e1005276. doi: 10.1371/journal.pgen.1005276 (PMC4457925; doi:10.1371/journal.pgen.1005276)
Supplement: S8 Table — cDNA was generated using SuperScript III Reverse Transcriptase (Life Technologies) according to the manufacturers protocol using random hexamer primers (Life Technologies). Quantitative PCR was performed as described in [66] with specific primers (listed in S9 Table). The relative mRNA levels were normalized to the housekeeping gene rrsA encoding 16S rRNA in E.coli. (PDF) [file pgen.1005276.s013.pdf]

Table S8 Verification of differentially expressed genes by RT-qPCR

**Sample**	**Expression dnaA[1,2]**	**Expression pka[1,2]**
MG1655	1	1
IF72	1.46 ± 0.023	1.22 ± 0.055

[1] Expression levels are normalized to the expression of rrsA
[2]The given values are the average expression in IF72 relative to MG1655.
