# Supplementary material for: The DnaA Protein Is Not the Limiting Factor for Initiation of Replication in Escherichia coli
Source: PLoS Genet. 2015 Jun 5;11(6):e1005276. doi: 10.1371/journal.pgen.1005276 (PMC4457925; doi:10.1371/journal.pgen.1005276)
Supplement: S9 Table — (PDF) [file pgen.1005276.s014.pdf]

Table S9 Primers used for RT-qPCR

Primer name	Primer sequence
pka-Fw	5`CATATTGCCGAACAGATTGG
pka-Rev	5`GCTGGACTTCATTGGCTGTA
pka-probe	5`TGCGTTCGCCGGATATTCCA
dnaA-Fw	5` GGCGAAAGAGCTGACTAACC
dnaA-Rev	5` CTCTCTTCACGCAACTGCTC
dnaA-probe	5` CATCGCCAATCTCCGGCAGA
rrsA-Fw	5`CGCAACCCTTATCCTTTGTT
rrsA-Rev	5`TAAGGGCCATGATGACTTGA
rrsA-probe	5` CTCCTTTGAGTTCCCGGCCG
